# Evidence that Bacteria Packaging by *Tetrahymena* Is a Widespread Phenomenon

**DOI:** 10.3390/microorganisms8101548

**Published:** 2020-10-07

**Authors:** Alicia F. Durocher, Alix M. Denoncourt, Valérie E. Paquet, Steve J. Charette

**Affiliations:** 1 Institut de Biologie Intégrative et des Systèmes, Pavillon Charles-Eugène-Marchand, Université Laval, Quebec City, QC G1V 0A6, Canada; alicia.durocher.1@ulaval.ca (A.F.D.); alix.denoncourt@gmail.com (A.M.D.); valerie.paquet@criucpq.ulaval.ca (V.E.P.); 2 Centre de Recherche de l’Institut Universitaire de Cardiologie et de Pneumologie de Québec, Hôpital Laval, Quebec City, QC G1V 4G5, Canada; 3 Département de Biochimie, de Microbiologie et de Bio-Informatique, Faculté des Sciences et de Génie, Université Laval, Quebec City, QC G1V 0A6, Canada

**Keywords:** bacteria, multilamellar bodies, *Tetrahymena*, fecal pellets, phagocytosis, exocytosis

## Abstract

Protozoa are natural predators of bacteria, but some bacteria can evade digestion once phagocytosed. Some of these resistant bacteria can be packaged in the fecal pellets produced by protozoa, protecting them from physical stresses and biocides. Depending on the bacteria and protozoa involved in the packaging process, pellets can have different morphologies. In the present descriptive study, we evaluated the packaging process with 20 bacteria that have never been tested before for packaging by ciliates. These bacteria have various characteristics (shape, size, Gram staining). All of them appear to be included in pellets produced by the ciliates *Tetrahymena pyriformis* and/or *T. thermophila* in at least one condition tested. We then focused on the packaging morphology of four of these bacteria. Our results demonstrated that, as shown previously for *Mycobacterium smegmatis*, the packaging of *Microbacterium oxydans*, *Micrococcus luteus*, and *Cupriavidus* sp. was formed of a single layer of material. The packaging of *Cellulosimicrobium* *funkei* was made of indistinguishable material. A different pellet morphology was obtained for each of the four bacterial strains studied. The ingestion of small bacteria resulted in rounder, denser, and more regular pellets. These results support the idea that bacteria packaging is a relatively widespread phenomenon.

## 1. Introduction

Protozoa are ubiquitous in many naturally occurring and human-made environments, such as cooling tower water, tap water, and pools, and on food in groceries [1,2,3]. A vast number of them prey on bacteria; however, some bacteria are able to resist predation by protozoa and survive digestion in the phagocytic pathway [4]. This can result in bacteria being included in fecal pellets expelled by protozoa, a phenomenon known as bacteria packaging. This process forms a typically spherical cluster of bacteria that can be covered by one or more layers of membrane, depending on the species of both bacteria and protozoa [1,5,6]. The packaging of different bacterial species by the same protozoan can give rise to different pellet morphologies; for instance, when *Tetrahymena pyriformis* feeds on *Mycobacterium smegmatis*, the pellets display very little membrane, whereas with *Campylobacter jejuni* the bacteria are surrounded by abundant membrane layers [7,8]. The pellet morphology also differs between protozoan species, as those produced by amoebae tend to contain fewer bacteria and have more membrane than those produced by ciliates [1,9].

Packaging confers many advantages to bacteria, such as resistance to some antibiotics and biocides; to low pHs; and to physical stresses, including sonication, desiccation, UV rays, and freeze–thaw cycles [1,10,11,12,13]. Packaging also enhances bacterial survival when exposed to long-term starvation [11,12]. It is also believed that packaging could help the propagation of pathogens through the air [14], as the diameter of a pellet, typically between 1 and 5 µm [9], falls within the range of respirable particles, which can penetrate lung alveoli and give rise to respiratory infections [15].

Bacteria packaging by *Tetrahymena* has mostly been studied for pathogens so far, including *Legionella pneumophila*, *Salmonella enterica*, *Escherichia coli* O157:H7, *C. jejuni*, and *Vibrio cholerae* [1,2,8,10,11]. Pathogenic bacteria are equipped with virulence factors that can facilitate resistance to digestion, but it has also been shown that at least some bacteria that are non-pathogenic to humans can be packaged by the social amoeba *Dictyostelium discoideum* [6]. Bacterial packaging could promote the survival of bacteria in harsh environments or act as a reservoir, as pellets can stay intact for weeks [11,12].

Ciliates can ingest bacteria at a very high rate [16] and quickly process particles through their digestive pathway. *T. pyriformis* in particular can ingest over 100% of its cell volume per hour [17]. This makes ciliates useful organisms with which to study bacteria packaging, as expelled pellets can be observed in the medium within less than an hour [18].

In the present study, we screened a group of 20 bacterial strains of various species which were previously shown to resist predation by the amoeba *D. discoideum* [6] for their capacity to be packaged by two ciliate species, *T. pyriformis* and *Tetrahymena thermophila*. All the bacterial strains were found in ciliate fecal pellets in at least one of the conditions tested. From this list, four strains were selected, and their packaging was further analyzed. Using different microscopy techniques, the pellets produced during these interactions were characterized in regard to the organization of bacteria within the pellets, the amount of material surrounding the bacteria, and the general appearance of the pellets.

## 2. Materials and Methods

### 2.1. Strains

The *Tetrahymena* strains used were *T. pyriformis* ATCC 30202, grown axenically in SPP medium at 25 °C (2% proteose peptone, 0.1% yeast extract, 0.2% dextrose, and 33 μM of FeCl_3_ supplemented with 250 μg/mL of streptomycin, 250 μg/mL of penicillin G, and 1.25 μg/mL of amphotericin B), and *T. thermophila* CU428.2, grown axenically in PP medium (2% proteose peptone and 10 μM of FeCl_3_ with the same antibiotics) at 30 °C [19,20]. The cells were subcultured when reaching confluence. Bacterial strains used in this study (Table 1) were a list of 20 bacterial strains previously identified to be resistant to *D. discoideum* predation [6]. Bacterial stock cultures were stored at −80 °C in Luria Broth (LB) medium (EMD, Mississauga, Canada) supplemented with 15% glycerol. Stock cultures were thawed and inoculated before each experiment on Tryptic Soy Agar (TSA) (EMD, Mississauga, Canada) plates, and incubated for 48h at 25 °C for bacteria from the environment or at 37 °C for those known to colonize warm-blooded animals or humans.

### 2.2. Co-Cultures

Co-culture assays were conducted in 6-well plates at a final volume of 3 mL. Co-cultures for the initial screening of the 20 bacteria were conducted in Tris-NaCl buffer (10 mM of Tris, 7 mM of NaCl, pH adjusted to 7.2–7.4) and incubated for 2–3 days, but were adjusted to the following conditions based on the results obtained in a similar experiment [7]. Bacteria grown for at least 48 h before the co-culture assay were suspended in Plate Count Broth (PCB) medium (0.5% yeast extract, 1% tryptone, and 0.2% dextrose) and the OD_595_ was adjusted to 1. *Tetrahymena* cells were transferred from their growth medium to PCB medium by centrifugations at 600× *g* for 2 min. A total of 3 × 10^5^ cells were added to each co-culture (one strain per co-culture), as well as 300 µL of one of the bacterial suspensions, for a final ratio of 1 ciliate:1000 bacteria in 3 mL of PCB. Co-cultures with *T. pyriformis* were incubated at 21 °C or 25 °C and those with *T. thermophila* were incubated at 21 °C or 30 °C (see Table 1), both for 4 h. Co-cultures were observed with an inverted microscope to confirm the presence of pellets before being processed for further analyses.

### 2.3. DAPI Staining

A 500 µL aliquot of each co-culture was fixed in the same volume of 8% paraformaldehyde (PFA), for a final concentration of 4%, and incubated for 30 min. PFA was replaced by centrifugation at 9600× *g* for 5 min with 40mM of NH_4_Cl in 1× PBS, followed by a second wash with 1× PBS. 1× PBS was replaced by 2.5 µg/mL of DAPI in 1× PBS, and the samples were incubated in the dark for 30 min, followed by 2 more washes in 1× PBS. The samples were spun down and the pellets were resuspended in 10–50 µL of 1× PBS depending on their size. A total of 10 µL of the sample was mixed with 10 µL of Prolong Gold (Invitrogen, Whitby, ON, Canada) on a microscope slide, and a coverslip was sealed on top. The samples were observed with a Zeiss Axio Observer Z1 microscope equipped with an Axiocam MRm camera (Carl Zeiss, North York, ON, Canada) in differential interference contrast (DIC) and epifluorescence. DAPI staining was reproduced 3 times for each bacterial species.

### 2.4. LIVE/DEAD Staining

The following experiment was only conducted with the four strains selected to further characterize the packaging: *M. oxydans*, *M. luteus*, *Cupriavidus* sp., and *C. funkei*. A total of 1 mL of 4 h co-culture was centrifuged for 2 min at 600× *g* and the supernatant containing the fecal pellets was collected. The supernatant was then centrifuged at 9600× *g* for 2 min and washed twice in 0.85% saline. A total of 1.5 µL of LIVE/DEAD bacterial viability stain (Life Technologies, Whitby, Canada) was added to the samples, followed by a 15 min incubation in the dark. The samples were pelleted and resuspended in 100–500 µL of saline depending on the pellet size. Then, 10 µL of these suspensions was placed on a microscope slide and a coverslip was sealed on top for immediate observation. The samples were observed with a Zeiss Axio Observer Z1 microscope equipped with an Axiocam MRm camera (Zeiss Canada, Dorval, QC, Canada). LIVE/DEAD staining was reproduced twice for each co-culture.

### 2.5. Transmission Electron Microscopy (TEM) Processing

This experiment was only conducted with the four selected bacterial strains. Samples from co-cultures, some incubated for more than 4 h to ensure maximal pellet retrieval, were fixed for 3 h in 0.1 M sodium cacodylate buffer (pH 7.3) containing 2% glutaraldehyde and 0.3% osmium tetroxide. After centrifugation, the sample pellets were resuspended in cooled molecular biology agarose (BioRad, Saint-Laurent, QC, Canada) diluted to 3% in PBS buffer. Once solidified, the samples were cut into cubes of about 3–5 mm and processed for TEM, as previously described [21].

## 3. Results

### 3.1. Co-Culture Assays

**Figure 1:**
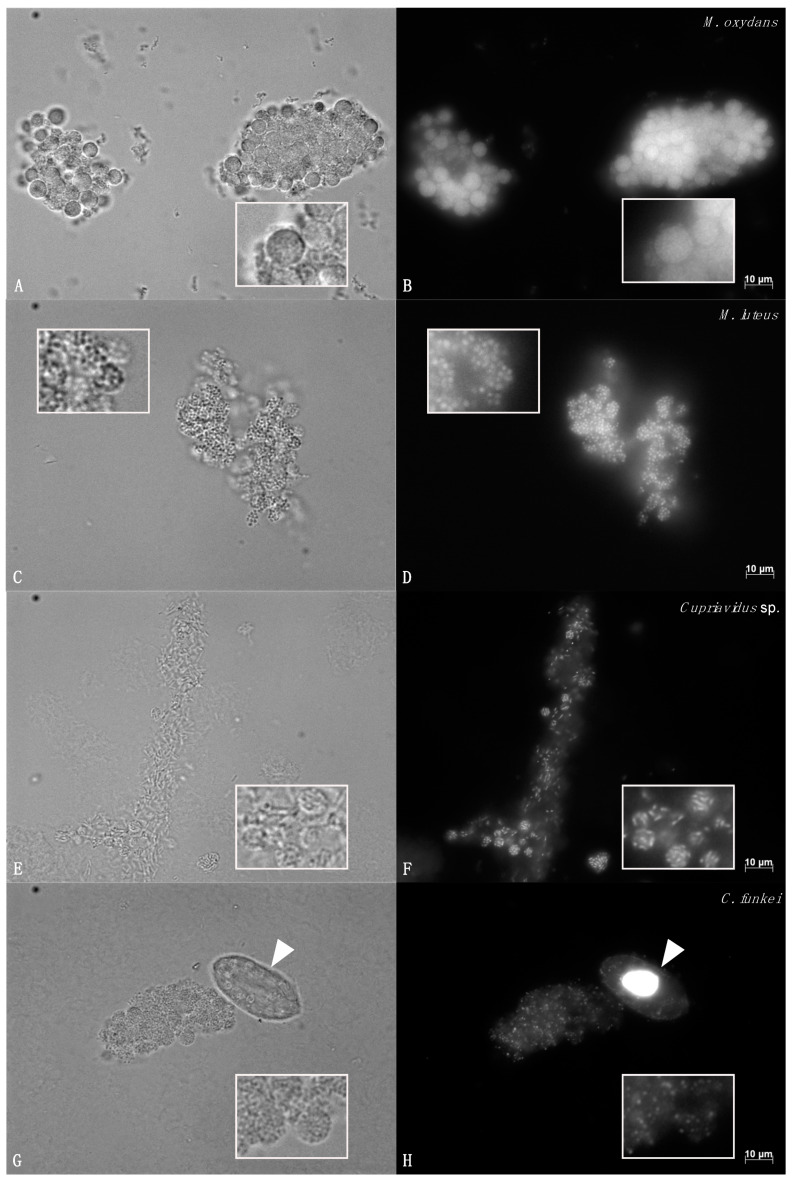
DIC and DAPI fluorescence microscopy of pellets produced by *T. pyriformis*. Pellets produced in a co-culture of *T. pyriformis* and *M. oxydans* (**A**,**B**), *M. luteus* (**C**,**D**), *Cupriavidus* sp. (**E**,**F**), and *C. funkei* (**G**,**H**). DIC images are shown in (**A**,**C**,**E**,**G**) and DAPI fluorescence images in (**B**,**D**,**F**,**H**). All the pictures were taken at 630× magnification. Scale bars: 10 µm. Inserts show a magnification of 2.25× to better see the pellets. Arrowheads indicate *T. pyriformis* cells.

Since resistance to digestion by protozoa is necessary for bacteria to be packaged [1,6], it is more likely to find bacteria resisting ciliate digestion among bacteria already known to resist digestion by other protozoa. It was observed that some bacterial species can be packaged by many different protozoa [5]. Consequently, in order to increase the possibility of identifying bacteria susceptible to packaging by *Tetrahymena* spp., this study focused on bacteria already known to resist predation from another protozoan. Our choice fell on a list of 20 bacterial strains known to resist *D. discoideum* amoeba predation [6]. These bacterial strains were co-cultured with *T. pyriformis* and *T. thermophila* in various conditions (Table 1) to assess whether they could also be packaged by ciliates. All the 20 bacteria were packaged in at least one of the conditions and tested based on DIC observation (Table 1). Each bacterial species was tested with either ciliate species at 21 °C or 25 °C for *T. pyriformis* and at 21 °C or 30 °C for *T. thermophila* (see Table 1 for details). The age of the bacterial culture was another condition considered, as the co-cultures were made with bacteria either 48 h after inoculation on TSA plates from a frozen culture or 14 days after inoculation. In the last case, the bacteria were incubated for 2 days at their optimal growth temperature (25 °C or 37 °C) and then for 12 days at 7 °C. These conditions (temperature and age of culture) were investigated to see whether they would influence pellet production; however, no general tendency was observed regarding the variation in pellet production. Pellets can be identified via DIC microscopy as spherical aggregates of bacteria with a characteristic relief, as illustrated in Figure 1.

DIC microscopy cannot confirm the presence of a membrane layer(s) surrounding packaged bacteria. This can be done using TEM. This type of microscopy also allows the analysis of the morphology of pellets [6,8]. *M. oxydans*, *M. luteus*, *C. funkei*, and *Cupriavidus* sp. were chosen to further characterize the packaging process by TEM. Since Gram-positive bacteria have so far been less studied in the context of packaging by protozoa, three of the four bacteria selected for the pursuit of this study fall into this group. These bacteria were also favored because of their different characteristics (Gram staining, cell shape, size), as presented in Table 2, and because of their ease of manipulation. Some bacteria, such as *Rhodococcus* spp. or *Cupriavidus necator*, were efficiently packaged but needed specific care compared to the other strains, and were thus discarded as candidates for this study to standardize the manipulations. The co-culture conditions used for the rest of the study were incubation at 25 °C with *T. pyriformis* or at 30 °C with *T. thermophila* with 48 h-old bacterial pre-cultures.

Pellets produced with each bacterial species had a distinct morphology which was consistent between both ciliate species (Figure 1 for *T. pyriformis* and data not shown for *T. thermophila*). The pellets produced with *M. oxydans* and *M. luteus* were round and regular. Those containing *M. oxydans* were densely packed with bacteria, while the *M*. *luteus* pellets were more loosely packed (Figure 1A–D). With *Cupriavidus* sp., the pellets were less regular and less tight and contained fewer bacteria (Figure 1E,F). With *C. funkei*, the pellets were round and regular and were similar to those containing *M. oxydans*, as seen in DIC microscopy (Figure 1G). However, the DAPI staining of packaged *C. funkei* bacteria revealed a diffuse fluorescent signal for the whole pellet, with a few bacteria producing a stronger signal (Figure 1H).

### 3.2. TEM Observations

**Figure 2:**
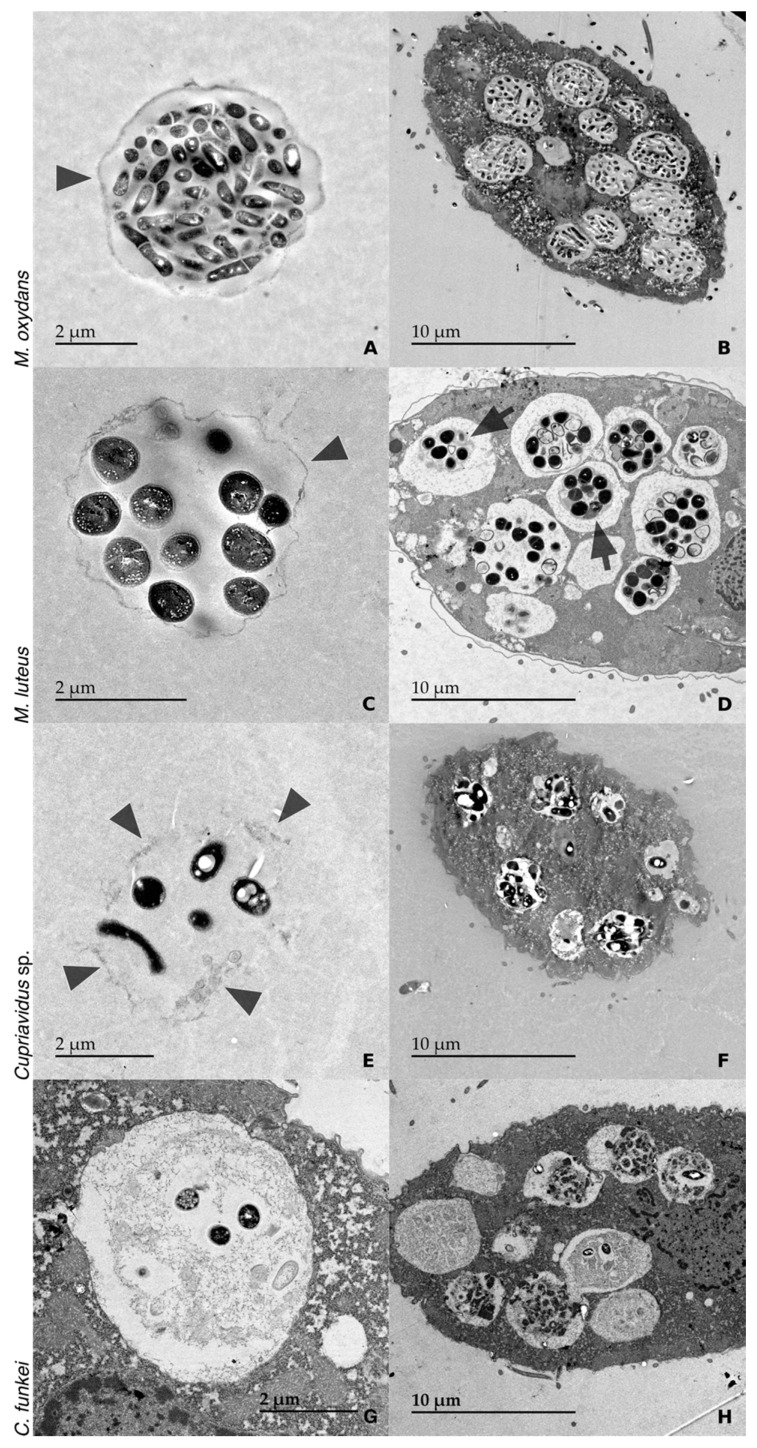
TEM images of bacteria packaging by *Tetrahymena*. Pellets produced in a co-culture of *T. pyriformis* and *M. oxydans* (**A**), *T. thermophila* and *M. luteus* (**C**), *T. thermophila* and *Cupriavidus* sp. (**E**), and *T. pyriformis* and *C. funkei* (**G**). In the case of *C. funkei*, since no expelled pellet was observed, a pellet in a late food vacuole prior exocytosis is shown. *T. pyriformis* containing *M. oxydans* (**B**), *T. thermophila* containing *M. luteus* (**D**), *T. thermophila* containing *Cupriavidus* sp. (**F**), and *T. pyriformis* containing *C. funkei* (**H**) are also shown. Co-cultures were performed for 4 h in all cases, except for *M. luteus*, where the co-culture lasted 16 h. Arrowheads point to the outer layer around the pellets when it is present. Multiple arrowheads are used when the layer is not continuous. Arrows point to thicker layers observed around the pellets in formation. Magnification: (**A**) 2500×; (**C**) 4000×; (**E**,**G**) 3000×. Pictures (**B**,**D**,**F**,**H**) were taken at 1000× magnification.

Figure 2 shows TEM images, which confirmed the morphology observed in DIC and epifluorescence microscopy. TEM micrographs were also used to determine the bacterial cell size (Table 2). With *M. oxydans*, the TEM images showed very dense and round pellets filled with bacteria and only one continuous membrane-like layer (Figure 2A). With *M. luteus*, the pellets were not as dense and regular. The membrane-like layer was thinner than the one observed with *M. oxydans* but was still continuous (Figure 2C). With *Cupriavidus* sp., the TEM images showed irregular and sparse pellets, and the material surrounding the bacteria was irregular and did not seem continuous (Figure 2E). With *C. funkei*, no excreted pellets were visible in TEM; however, samples from the same experiment observed in DIC, and epifluorescence microscopy showed numerous external pellets. However, *C. funkei* pellets still transiting within the protozoa were observed in TEM (Figure 2G,H). Only a few intact bacteria remained, surrounded by partially digested ones. This is in agreement with the epifluorescence observations, which showed only a few brightly fluorescent bacteria in faintly fluorescent pellets, possibly resulting from the partially digested bacterial cell remains.

Pellets from all four bacterial species were detected in the *Tetrahymena* phagocytic vesicles (Figure 2). Intracellular pellets containing *M. oxydans* bacteria were similar to the expelled ones (Figure 2B). For *M. luteus*, a thick material of an undefined nature was surrounding packaged bacteria inside the *Tetrahymena* vesicles (Figure 2D and Figure S1). The vacuoles of *Tetrahymena* co-cultured with *Cupriavidus* sp. contained structures similar to the expelled pellets, with little membrane and being sparsely filled with bacteria (Figure 2F). Finally, for *C. funkei* the intracellular pellets seemed to have little to no material surrounding the bacteria (Figure 2H).

### 3.3. LIVE/DEAD Assay

**Figure 3:**
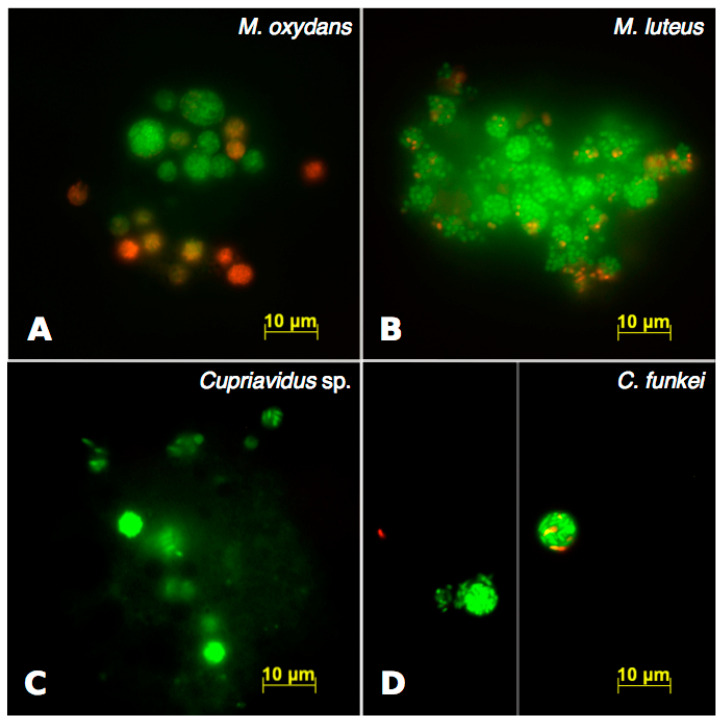
Bacterial cells are viable in pellets produced by *Tetrahymena*. Pellets produced in a co-culture of *T. pyriformis* and *M. oxydans* (**A**), *M. luteus* (**B**), or *Cupriavidus* sp. (**C**). Pellets produced in a co-culture of *T. thermophila* and *C. funkei* (**D**). Panel (**D**) is a composite of 2 different images, as no single image contained more than one pellet. Samples were stained with LIVE/DEAD bacterial viability stain. All the pictures were taken at 630× magnification and are merge images of SYTO 9 (green; viable cells) and propidium iodide (red; dead cells) fluorescence.

LIVE/DEAD staining revealed that, for each bacterial species, most of the packaged bacteria were viable (green) (Figure 3). For *M. oxydans*, some pellets contained almost exclusively viable bacteria, while others mainly contained damaged ones (red).

## 4. Discussion

This study demonstrated the phenomenon of bacteria packaging by *Tetrahymena* and demonstrates the packaging of several new bacterial species. Our study adds to the growing list of bacteria susceptible to packaging by protozoa, which now includes many non-pathogenic species. Our findings also demonstrate that the nature and morphology of pellets may vary appreciably between bacterial strains.

A specific pellet morphology was identified for each bacterial strain (Figure 1, Figure 2 and Figure 3). There were no obvious differences in pellet morphology between the pellets produced by *T. pyriformis* and *T. thermophila*, suggesting that the pellet morphology is more influenced by the bacteria consumed than the protozoa, at least for the two ciliates used in this study.

With *M. oxydans*, a small Gram-positive bacillus, the pellets were very dense, regular, and round, packed with seemingly intact bacteria surrounded by a continuous membrane-like layer. Very few bacteria appeared to be digested based on the LIVE/DEAD staining. TEM also showed intact, sometimes dividing cells in the pellets, with a small amount of membrane-like material surrounding the structure. It was reported that *M. oxydans* is not efficiently digested by *T. pyriformis* and is not its preferred prey [23], which could explain why it was barely degraded by both ciliates in the present study.

As with *M. oxydans,* the pellets produced with *M. luteus* were quite round and regular, but the continuous material surrounding the bacterial cells was thinner than that surrounding the *M. oxydans* cells. Intracellular pellets appeared to have a thicker membrane-like material surrounding them (Figure S1) and more partially digested bacteria than the ejected pellets. Pellets containing damaged bacteria might be less sturdy than those containing mainly intact bacteria, and therefore fall apart once ejected.

With *Cupriavidus* sp., the pellets were irregular and sparse. These pellets contained scattered bacteria enveloped by chunks of material rather than a continuous layer. *Cupriavidus* sp. was the only Gram-negative bacteria tested in this study and generated a quite different pellet morphology compared to the other bacteria. This strain of *Cupriavidus* sp. contains intracellular inclusions and was isolated as part of a biofilm, unlike the other three bacteria [22]. These characteristics may influence the pellet morphology.

Pellets containing *C. funkei* were very similar to the ones produced with *M. oxydans*: round, regular, and filled with bacteria. However, the DAPI staining showed that they contained only a few intact *C. funkei* cells, surrounded by a diffuse fluorescent signal suggesting bacterial degradation. No ejected pellets were seen in TEM, but packaged bacteria were present in late-stage vacuoles, ready to be ejected via exocytosis (Figure 2H). Phagocytic vesicles are considered late-stage vacuoles when the membrane does not touch its contents [24]. Pellets in those residual vacuoles are assumed to have the same morphology as ejected pellets, as no more digestive activity can likely affect them [25,26]. Since the centrifugation steps performed during the preparation of TEM samples may damage fragile, expelled pellets, the contents of late-stage vacuoles may be a more reliable way to analyze the pellet morphology. TEM observations confirmed that only a few *C. funkei* cells remained whole in late-stage vacuoles and that the rest of the pellet was made of either partially digested, compacted bacteria or unidentified material. In both cases, the pellets lacked a surrounding membrane-like layer, suggesting that *C. funkei* could be compacted rather than packaged, with the digested cells “protecting” the seemingly intact ones. A similar phenomenon of particle compaction in vacuoles was previously reported for *T. pyriformis*-fed multilamellar bodies produced by *D. discoideum* amoeba [18].

While *C. funkei* and *M. oxydans* are both Gram-positive rods, *C. funkei* seems to be more digestible than *M. oxydans*, judging by the numerous degraded *C. funkei* cells in *Tetrahymena* digestive vacuoles. This might explain the difference in pellet morphology between those species. While all four bacterial strains could resist digestion to some extent, some were more resistant than others. For highly resistant, non-digestible bacteria like *M. oxydans*, it was suggested that the material used in the formation of pellets comes from dead cells already present in the inoculum [1].

Although considerably different, *M. oxydans* and *M. luteus* pellets were the most similar out of the ones analyzed in this study. Both bacteria are small and Gram-positive, but their cell shape differs.

The LIVE/DEAD observations yielded interesting results. For all the bacterial strains except *M. oxydans*, the pellets mostly contained intact bacteria with a few damaged cells per pellet. On the other hand, most *M. oxydans* pellets contained either almost exclusively intact or damaged bacteria. It was suggested by Thurman et al. that bacteria present in high numbers in vacuoles are more likely to evade digestion than bacteria present in low numbers [27]. As seen in Figure 3A, the “all intact” pellets were generally bigger than the “all damaged” pellets. Although these “all damaged” pellets contained a lot more bacteria than the numbers reported in the previous study, who found that the contents of vacuoles containing 6 cells or less were digested with the most efficiency, the same tendency was observed [27]. However, the bacterial species tested in Thurman et al. may be more digestible than *M. oxydans*. Even when *M. oxydans* cells were damaged, they still mostly kept their shape and were not entirely digested, as they were expelled inside pellets. This indicates that while *M. oxydans* may be killed by *Tetrahymena*’s digestive process, it cannot be efficiently digested. This could explain why *T. pyriformis* exhibits a low grazing preference for *M. oxydans* in a multispecies biofilm [23].

The *C. funkei* LIVE/DEAD results were inconsistent with the TEM observations. LIVE/DEAD staining showed that most of the bacterial cells inside pellets were intact, while TEM showed that most of the cells were digested and only a few cells per pellet remained whole. The TEM observations seem to be more reliable here, as it was difficult to observe many pellets in LIVE/DEAD samples. The staining procedure for LIVE/DEAD requires the centrifugation of non-fixed samples, which may damage the pellets, while abundant still-in-transit pellets could be analyzed in TEM. It is possible that the pellets observed in the LIVE/DEAD samples were the contents of early digestive vacuoles from lysed *Tetrahymena* cells, which are also sensitive to high-speed centrifugation. A diffuse fluorescent signal was often observed surrounding the *C. funkei* “pellets” in LIVE/DEAD experiments, which could be cytoplasmic content.

The present study shows that bacteria packaging is a widespread phenomenon and is not restricted to bacteria pathogenic to humans. While previous studies have focused on the packaging of Gram-negative bacteria and mycobacteria, our findings demonstrate that Gram-positive bacteria can also be packaged by protozoa.

## 5. Conclusions

The results of this descriptive study demonstrate that a large number of bacterial species can be packaged by *Tetrahymena*. In addition, we show that ciliates can package non-pathogenic bacteria, in contrast to previous studies which have mainly focused on human pathogenic strains. Pathogenic bacteria are typically packaged with multiple layers of membrane [1,8]. Interestingly, as previously reported with *M. smegmatis* [7], this study shows that pellets containing non-pathogenic bacteria display a more simple structure than those produced with pathogenic bacteria, being composed of one membrane-like layer or undefined covering material.

It would be interesting to quantify the digestibility of the bacteria and how it relates to the pellet morphology in further works. Future studies should also consider the impact of the protozoa:bacteria ratio in co-cultures on pellet production.

## Figures and Tables

**Table 1:** Amoeba-resistant bacteria packaged by ciliates. Various non-pathogenic bacteria presenting an amoeba-resisting phenotype, as identified by Paquet et al., 2016, were co-cultured with two ciliate species. The production of bacteria-containing pellets was quantified by observing 10 fields at random on a microscope slide and counting the visible pellets. The extent of pellet production of each co-culture based on DIC observations is graded as follows: counts of 20+ visible pellets were considered excellent pellet production (++++, dark green); 10–19 pellets were considered good (+++, light green); 3–9 pellets were considered intermediate (++, yellow); and 0–2 pellets were considered mediocre (+, orange). If no pellet was visible in the initial co-culture upon observation with an inverted microscope (-, red), it was considered that there was no pellet production and no slide was further prepared. Thus, the classifications from excellent to mediocre all yielded some pellets that were visible in the co-culture before sampling. Bacteria in bold were chosen for the rest of the pellet characterization.

Bacteria	Source	*T. thermophila* ^1^	*T. pyriformis* ^2^
*Cupriavidus basilensis*	Isolated from drinking water pipes [22]	+	++
***Cupriavidus*** **sp.**	Isolated from drinking water pipes [22]	+	++
*Micrococcus luteus*	Janet Martha Blatny (NTNU ^3^, Norway)	++	+++
*Micrococcus luteus* US4	USDA	+	+
*Rathayibacter tritici* LBUM-572	Martin Filion (Moncton University, Canada)	+	++
*Rhodococcus erythropolis* US1	USDA	++	+
*Rhodococcus erythropolis* US2	USDA	+	+
*Rhodococcus fascians* US1	USDA	++	++
*Rhodococcus fascians* US2	USDA	+	++
*Cupriavidus necator* US1	USDA	+	+++
*Duganella zoogloeoides* LBUM-382	Martin Filion (Moncton University, Canada)	+	+
*Kocuria kristinae*	Janet Martha Blatny (NTNU, Norway)	++	+++
***Microbacterium oxydans* US1**	USDA	++++	++++
***Micrococcus luteus***	Pierre Cosson (Geneva University, Switzerland)	++	+
*Micrococcus luteus* 8_4_14 X2	Janet Martha Blatny (NTNU, Norway)	+	+
*Micrococcus luteus* D_1_6 X2	Janet Martha Blatny (NTNU, Norway)	-	++++
*Micrococcus luteus* US3	USDA	+	+++
*Rhodococcus erythropolis*	Janet Martha Blatny (NTNU, Norway)	+	++++
*Rhodococcus pyridinivorans*	Janet Martha Blatny (NTNU, Norway)	+	++
***Cellulosimicrobium funkei***	Janet Martha Blatny (NTNU, Norway)	+++	++

^1^*T. thermophila,* co-cultures at 21 and 30 °C were tested. ^2^ *T. pyriformis,* co-cultures at 21 or 25 °C were tested. ^3^ Norwegian University of Science and Technology.

**Table 2:** Characteristics of the four bacteria studied.

Bacteria	Gram	Shape	Size ^1^ (µm)
*Microbacterium oxydans* US1	+	Rod	1 × 0.25
*Micrococcus luteus*	+	Coccus	0.5
*Cellulosimicrobium funkei*	+	Rod	1 × 0.5
*Cupriavidus* sp.	-	Rod	2 × 0.5

^1^ TEM micrographs were used to determine the bacterial cell size.

## Supplementary Materials

The following are available online at https://www.mdpi.com/2076-2607/8/10/1548/s1: Figure S1: the material surrounding M. luteus pellets in formation is thick and of an undefined nature.
