# Supplementary material for: Evidence that Bacteria Packaging by Tetrahymena Is a Widespread Phenomenon
Source: Microorganisms. 2020 Oct 7;8(10):1548. doi: 10.3390/microorganisms8101548 (PMC7601845; doi:10.3390/microorganisms8101548)
Supplement: Supplementary file 1 [file microorganisms-08-01548-s001.pdf]

SUPPLEMENTARY FILE

Evidence that bacteria packaging by Tetrahymena is a widespread phenomenon

Alicia F. Durocher[1,2,3], Alix M. Denoncourt[1,2,3], Valérie E. Paquet[1,2,3]

and Steve J. Charette[1,2,3]*

1. Institut de Biologie Intégrative et des Systèmes, Pavillon Charles-Eugène-Marchand, Université Laval, Quebec City, QC, Canada

2. Centre de recherche de l'Institut universitaire de cardiologie et de pneumologie de Québec, Hôpital Laval, Quebec City, QC, Canada

3. Département de biochimie, de microbiologie et de bio-informatique, Faculté des sciences et de génie, Université Laval, Quebec City, QC, Canada

*Corresponding author:

Steve J. Charette, 1030 avenue de la Médecine, Pavillon Marchand, local 4245, Université Laval, Quebec City, QC, Canada, G1V 0A6, telephone: 1-418-656-2131, ext. 406914, fax: 1-418-656-7176, email: steve.charette@bcm.ulaval.ca

Figure S1 illustrates how some still-transiting pellets inside *Tetrahymena* cells co-cultivated with *M. luteus* had a thicker membrane layer surrounding bacterial cells than expelled *M. luteus* pellets. This difference could be due to pellets being partly degraded once they are expelled from ciliate cells.

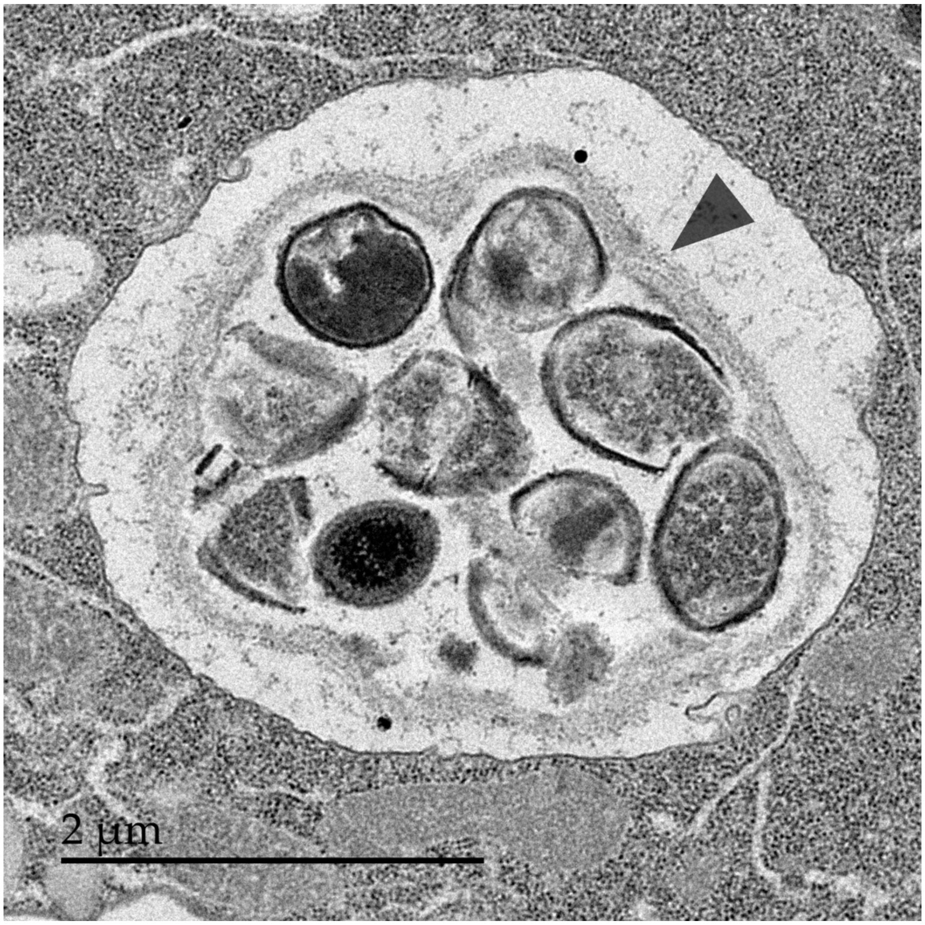

Figure S1. The material surrounding *M. luteus* pellets in formation is thick and of undefined nature. A *T. thermophila* vacuole containing a *M. luteus* pellet still in formation. An arrowhead points the thick outer layer. Picture taken at 5000X magnification.
